# An Overview on Fecal Profiles of Amino Acids and Related Amino-Derived Compounds in Children with Autism Spectrum Disorder in Tunisia

**DOI:** 10.3390/molecules28073269

**Published:** 2023-04-06

**Authors:** Mariem Chamtouri, Abderrahmen Merghni, Nuria Salazar, Begoña Redruello, Naoufel Gaddour, Maha Mastouri, Silvia Arboleya, Clara G. de los Reyes-Gavilán

**Affiliations:** 1 Department of Microbiology and Biochemistry of Dairy Products, Instituto de Productos Lácteos de Asturias (IPLA-CSIC), 33300 Villaviciosa, Spain; 2 Laboratory of Transmissible Diseases and Biologically Active Substances LR99ES27, Faculty of Pharmacy, University of Monastir, Monastir 5000, Tunisia; 3 Laboratory of Antimicrobial Resistance LR99ES09, Faculty of Medicine of Tunis, University of Tunis El Manar, Tunis 1068, Tunisia; 4 Diet, Microbiota, and Health Group, Instituto de Investigación Sanitaria del Principado de Asturias (ISPA), 33011 Oviedo, Spain; 5 Scientific and Technical Services, Instituto de Productos Lácteos de Asturias (IPLA-CSIC), 33300 Villaviciosa, Spain; 6 Unit of Child Psychiatry, Monastir University Hospital, Monastir 5000, Tunisia

**Keywords:** autism, amino acids, biogenic amines, neurotransmitters, glutamate, alanine, GABA, feces

## Abstract

Autism spectrum disorder (ASD) is a neurodevelopmental pathology characterized by the impairment of social interaction, difficulties in communication, and repetitive behaviors. Alterations in the metabolism of amino acids have been reported. We performed a chromatographic analysis of fecal amino acids, ammonium, biogenic amines, and gamma aminobutyric acid (GABA) in Tunisian autistic children from 4 to 10 years, and results were compared with their siblings (SIB) and children from the general population (GP). ASD presented significantly higher levels of fecal amino acids than SIB and GP; differences being more pronounced in younger (4–7 years) than in older (8–10 years) individuals whereas no changes were found for the remaining compounds. Lower levels of histidine were the only difference related with severe symptoms of autism (CARS scale). A linear discriminant analysis (LDA) based on fecal amino acid profiles clearly separated ASD, SIB, and GP at 4 to 7 years but not at more advanced age (8–10 years), evidencing more pronounced alterations in younger children. The relationship of fecal amino acids with autism needs deeper research integrating blood analytical parameters, brain metabolism, and intestinal microbiota. Fecal amino acids could be targeted for designing personalized diets to prevent or minimize cognitive impairments associated with ASD.

## 1. Introduction

Autism Spectrum Disorder (ASD) comprises a set of neurodevelopmental dysfunctions beginning at early childhood and characterized by an impairment in social communication and behavioral problems, such as repetitive conducts and restricted patterns of interest [1]. The reported prevalence of ASD has dramatically increased in recent decades, being recently estimated as 1% worldwide [2]. Although a relevant part of cases could be attributed to the improvement of diagnostic criteria and increased surveillance and awareness, the importance of ASD cannot be neglected. ASD depends on genetic predisposition; although, scientific evidence points to some environmental factors also playing a key role in the disease, including prenatal or postnatal exposure to chemicals and drugs, pollution, stress, maternal infections, epigenetic influences, immune abnormalities, and dietary patterns [3].

The concept microbiota–gut–brain axis has emerged in past years to describe the interactions between these three systems; which, although multifactorial and complex, involve several ways of communication between the gastrointestinal microbiota and brain [4]. Gastrointestinal problems (such as diarrhea, constipation, bloating and gastroesophageal reflux) are frequently present in individuals diagnosed with ASD. Associated with such intestinal problems, alterations in the composition and metabolic activity of the intestinal microbiota as well as in some metabolites in serum, feces, and urine have also been reported [5,6]. Although the mechanisms involved are still unknown, it has been hypothesized that alterations in the gut microbiota of children with ASD may lead to an unbalance of certain metabolites that are known to be important in the microbiota–gut–brain communication. Additionally, dysbiosis of the intestinal microbiota is often associated with a disruption of the intestinal mucosal barrier, increasing gut permeability to neurotoxic compounds from dietary origin or generated by the action of intestinal bacteria; which, leads to the disturbance of neuroregulatory mechanisms and to the impairment of the normal brain development [5]. In this regard, alterations in fecal levels of ammonium and several amino acids, some of them acting as or being precursors of, neurotransmitters, as well as bacterial-derived aromatic amino acid metabolites such as p-cresol and 4-ethylphenol, have been associated with ASD [6,7,8].

Despite as indicated just above, there are still few studies leading to the association of fecal amino acids and microbial amino-derived metabolites, such as biogenic amines or some neurotransmitters, with the severity and progression of ASD. In addition, the possible contribution of geographical location, local traditions, contaminant levels, age, and dietary patterns, among others, remain largely unexplored. In this context, the aim of the present study was to determine the possible alterations in fecal amino acids, ammonium, and some amino-derived compounds in Tunisian autistic children, as compared with a group of siblings and of children from the general population. The secondary aim was to assess the potential of fecal amino acids to differentiate ASD children from the general population.

## 2. Results

### 2.1. Subjects Characteristics

A total of 74 children (aged 4 to 10 years) were enrolled in the city of Monastir (Tunisia) for this study. Twenty-eight were autistic children (ASD group), 18 were brothers and sisters of autists (siblings; SIB group) matched by age, and 28 were children from the general population matched by age and gender. General characteristics of the sample population as well as the degree of severity of autism of participants according to the CARS score are summarized in Table 1. Most of the children diagnosed with ASD were male, mostly in the group of age 8–10 years. According to the CARS score, 11 autistic children (39.3%) presented a mild to moderate degree of autism (score range 30–36) whereas 17 (60.7%) were diagnosed as severe (score 37–60).

### 2.2. Profiles of Fecal Ammonium, Free Amino Acids, and Amino-Derived Compounds

**Figure 1:**
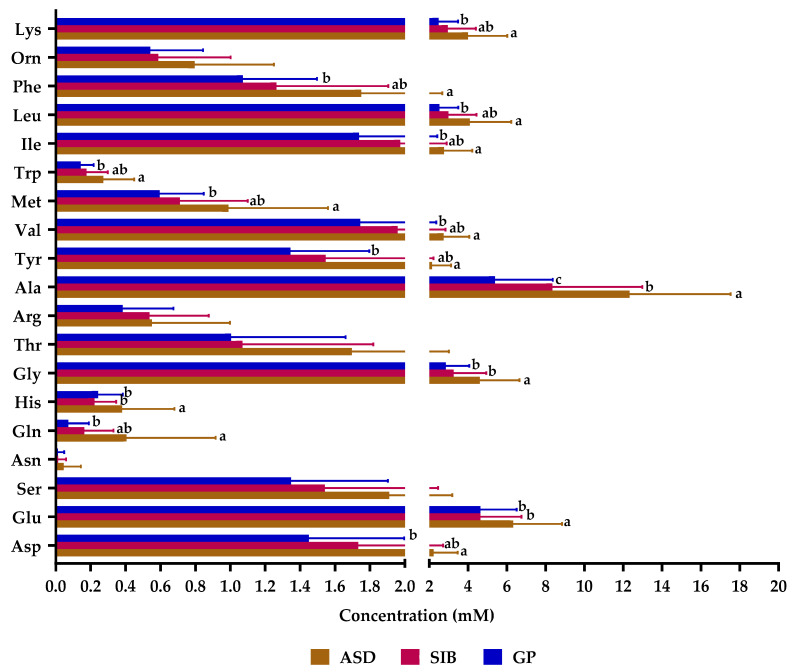
Fecal levels of amino acids in samples from autistic children (ASD), their siblings (SIB), and children from the general population (GP) in Tunisia. Asp: aspartate; Glu: glutamate; Ser: serine; Asn: asparagine; Gln: glutamine; His: histidine; Gly: glycine; Thr: threonine; Arg: arginine; Ala: alanine; Tyr: tyrosine; Val: valine; Met: methionine; Trp: tryptophan; Ile: Isoleucine; Leu: leucine: Phe: phenylalanine; Orn: Ornithine; Lys: lysine. Bars represent mean values and horizontal lines on the bars represent standard deviation. Different letters (a, b, ab, c) indicate significant differences among groups of children for each amino acid (*p* < 0.05).

From the compounds analyzed, proline, tryptamine, and phenylethylamine were under the limit of detection of the chromatographic technique and were not considered further in our analyses. Fecal samples from the ASD group contained significantly higher levels of total amino acids and of each of the different subgroups of amino acids (branched chain: valine, leucine and isoleucine; aromatic: tyrosine, phenylalanine and tryptophan; and aliphatic: alanine, valine, leucine, isoleucine, and glycine) than the feces from SIB and GP groups (Table S1). Alanine, glutamate, and glycine were the most abundant, followed by leucine and lysine (Figure 1). All individual amino acids were at higher levels in samples from the ASD groups and the differences between ASD and GP reached statistical significance for most of them, with the exception of serine, asparagine, threonine, arginine, and ornithine. In addition, levels of glutamate, histidine, glycine, and alanine were significantly higher in samples from ASD children as compared to SIB, with the levels of asparagine, glutamine, alanine, tyrosine, valine, methionine, isoleucine, leucine, and phenylalanine also differing significantly between the group of SIB and GP. Notably, the ratio of alanine to glutamate showed a significantly increasing trend in the direction GP < SIB < ASD (1.159 ± 0.544 mM; 1.738 ± 0.588 mM; 1.989 ± 0.583 mM; *p* < 0.05) as it also occurred, although at lower rates, for most of the other amino acids with respect to glutamate in samples of GP as regards to ASD. This suggests a disbalance between fecal levels of most amino acids as related to glutamate in autism.

No significant differences were found for the biogenic amines agmatine, histamine, tyramine, putrescine, and cadaverine, and the neurotransmitter gamma-aminobutyric acid (GABA) among samples of the three groups of children (Figure S1). In spite of this, ammonium showed a trend of presenting higher levels in the group of ASD, with respect to SIB, and in this group with respect to GP, without reaching statistical significance.

Our results indicate clear alterations in the profile of fecal amino acids in children suffering ASD as compared with the individuals from SIB and GP groups.

### 2.3. Alteration of Fecal Free Amino Acids Profiles as a Function of Age

**Figure 2:**
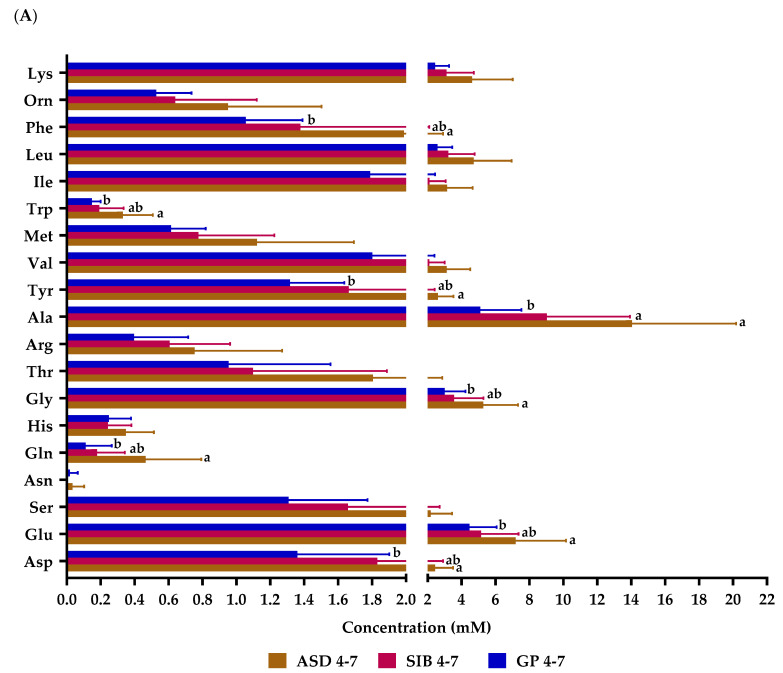

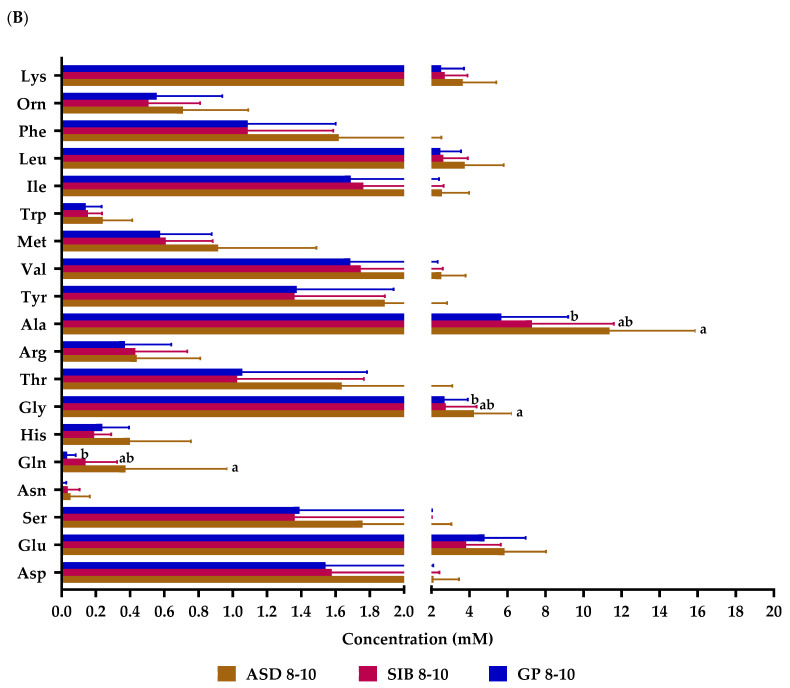
Fecal levels of amino acids in samples from autistic children (ASD), their siblings (SIB), and children from the general population (GP) in Tunisia stratified by age: (**A**) 4–7 years, (**B**) 8–10 years. Asp: aspartate; Glu: glutamate; Ser: serine; Asn: asparagine; Gln: glutamine; His: histidine; Gly: glycine; Thr: threonine; Arg: arginine; Ala: alanine; Tyr: tyrosine; Val: valine; Met: methionine; Trp: tryptophan; Ile: Isoleucine; Leu: leucine: Phe: phenylalanine; Orn: Ornithine; Lys: lysine. Bars represent mean values and horizontal lines on the bars represent standard deviation. Different letters (a, b, ab) indicate significant differences among groups of children for each amino acid (*p* < 0.05).

Levels of fecal amino acids were separately analyzed in children from 4 to 7 years of age, and in children from 8 to 10 years. This division was established considering that at the age 4 to 7 years, most autistic children in Tunisia remain at home; whereas, between 8 to 10 years, most children assist or are institutionalized in specialized Centers. At the age of 4–7 years, significantly higher levels of total, aromatic, and aliphatic amino acids (Table S1), as well as of the individual amino acids aspartate, glutamate, glutamine, glycine, alanine, tyrosine, tryptophan, and phenylalanine, were found in ASD with respect to GP group (Figure 2). In contrast, at the age of 8–10 years, only the group of aliphatic amino acids (Table S1), and the individual amino acids glutamine, glycine, and alanine, displayed significantly higher concentration in the feces of ASD than in those of GP.

No differences were found in fecal levels of biogenic amines, ammonium, and GABA among the three groups of children at the two age ranges considered (Figure S2).

These results evidence considerably more pronounced alterations in the amino acids profile between ASD and GP in younger individuals than at more advanced age.

### 2.4. Influence of the Severity of the Disease in the Profile of Fecal Free Amino Acids in Autistic Children

**Figure 3:**
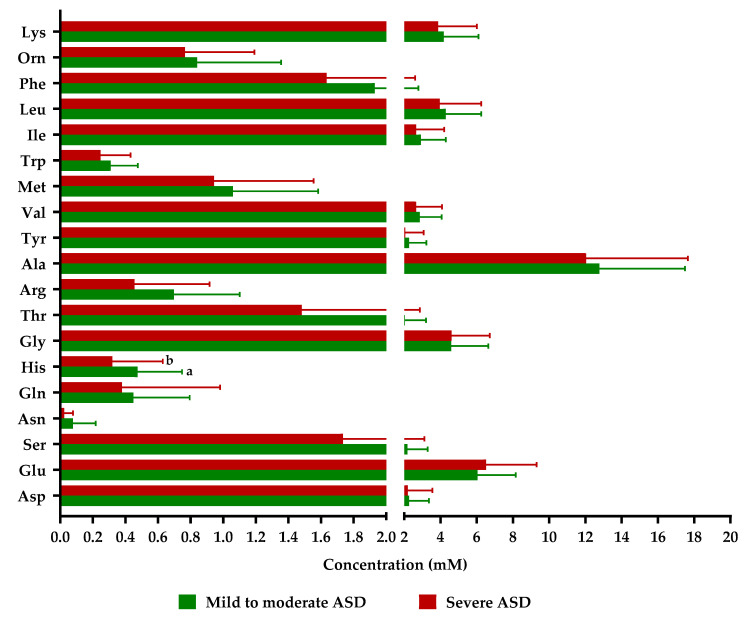
Fecal levels of amino acids in samples from autistic children (ASD), stratified by severity of the disease according to CARS score: mild to moderate (30–36) and severe (37–60). Asp: aspartate; Glu: glutamate; Ser: serine; Asn: asparagine; Gln: glutamine; His: histidine; Gly: glycine; Thr: threonine; Arg: arginine; Ala: alanine; Tyr: tyrosine; Val: valine; Met: methionine; Trp: tryptophan; Ile: Isoleucine; Leu: leucine: Phe: phenylalanine; Orn: Ornithine; Lys: lysine. Bars represent mean values and horizontal lines on the bars represent standard deviation. Different letters (a, b) indicate significant differences between groups of children for each amino acid (*p* < 0.05).

We stratified the group of ASD children according to the severity of the disease following the CARS scale (mild to moderate, and severe) and analyzed differences in metabolites between the two subsets. A trend to lower fecal levels in subjects with a severe degree of the disease was found for most but not all compounds; histidine being the sole case displaying significantly lower concentrations in individuals with severe symptoms (Figure 3 and Figure S3).

### 2.5. Grouping Children According to Fecal Free Amino Acid and Amino-Derived Compounds as Depending on Age

**Figure 4:**
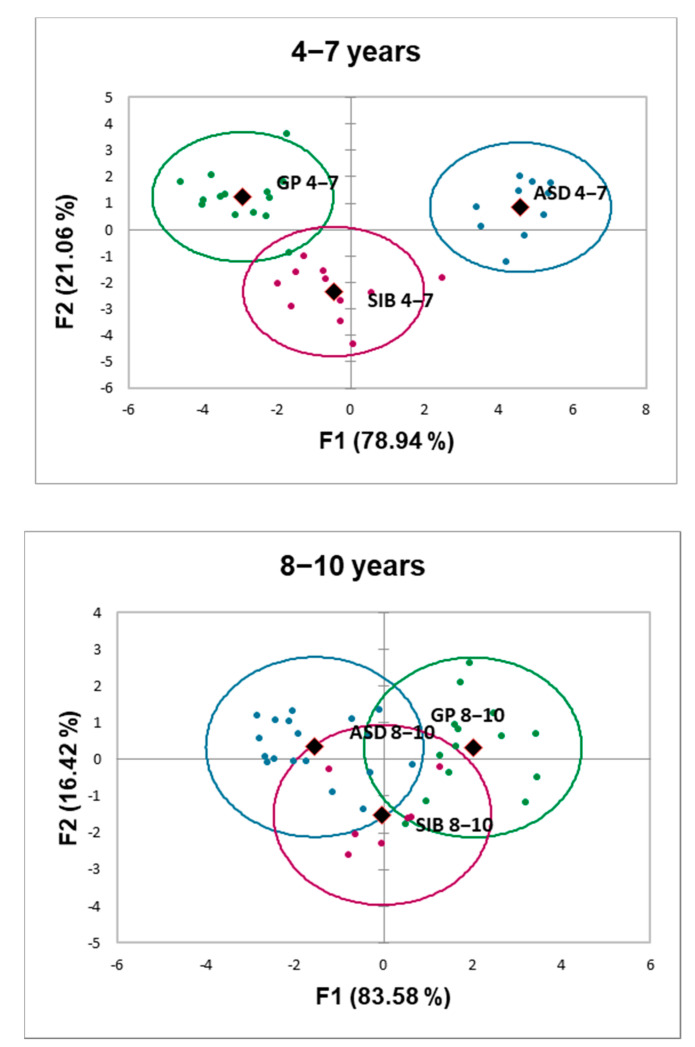
Projection of the metabolic profiles of the three groups of children stratified by age (4–7 years, and 8–10 years) into the plane formed by the two classification functions (F1 and F2) obtained from the linear discriminant analysis based on fecal levels of 19 protein amino acids excluding proline and cysteine and including the non-protein amino acid ornithine. Blue spots represent autistic children: ASD; green spots represent individuals from the general population (GP); purple spots represent the siblings from autistic children (SIB): Oval lines around individual spots are arbitrarily representing groups based on the profile of compounds analyzed. Centroids are indicated with rhomboid shapes. Percentage of variance explained by each of the two functions is indicated within parentheses in each axis.

A linear discriminant analysis (LDA) was performed to evaluate whether the fecal metabolic profiles were useful to discriminate the three groups of children established in our study: ASD, SIB, and GP. For this purpose, the concentration of free amino acids present in feces were analyzed separately for each range of age: 4–7 and 8–10 years (Figure 4).

At the age of 4–7 years, the LDA returned two canonical functions: F1 explained 78.94% of variance (eigenvalue 10.391) and F2 explained 11.34% of variance (eigenvalue 2.772). The correlations of variables to discriminant functions (Table S2) indicated that the main contributors to F1 in a decreasing order were alanine (coefficient: 0.669), tyrosine (coefficient: 0.662), glutamine (0.599), tryptophan (0.549), glycine (0.536), phenylalanine (0.536), and valine (0.531). The main contributors to F2 were asparagine (coefficient: 0.259) and histidine (coefficient: 0.143). Figure 4 plots F1 vs. F2. The first function clearly separated members of the ASD group from members of the other two groups; whereas, F2 provided a good separation between GP and SIB, with only one member of the GP group being clustered with SIB. On the other hand, considering the means of metabolic parameter values for each group, the probability of individuals for being allocated in the correct group was 100% for ASD and SIB and 92.86% for GP.

At the age of 8–10 years, discriminant functions explained 83.58% of variance for F1 (eigenvalue 2.771) and 16.42% of variance for F2 (eigenvalue 0.545), respectively. The correlations of variables to functions are summarized in Table S3. The main contributors at that age to F1 and F2 were alanine in FI (coefficient: −0.624) and in F2 glutamate (coefficient: 0.47). Not a good separation among groups was obtained with any of the functions and, in fact, the probability of individuals for being clustered in the correct group decreased to 57.14% in the GP group, 55.56% in the ASD group, and 0.00% for SIB.

LDA evidenced that the three groups of children considered can be clearly separated based on the fecal profile of amino acids at ages of 4–7 years. Later on, these metabolites did not provide a good discrimination, suggesting that the early differentiation of ASD based on the fecal profile of amino acids is progressively attenuated as age advances.

## 3. Discussion

ASD is one of the most severe neurodevelopmental conditions worldwide and its prevalence has considerably increased in recent years. The microbiota–gut–brain axis has recently emerged as an important keystone factor in neurodevelopmental disorders, and it is known that intestinal dysbiosis can lead to an unbalance of metabolites that can affect the gut–brain communication. Nevertheless, there is still not too much information about alterations in the fecal levels of amino acids, amino-derived compounds, ammonium, and some neurotransmitters in ASD children, and how these alterations could be related with disease severity. To our knowledge, this work entails the first observational study determining the association between those metabolites and autism in feces of Tunisian children. Autistic children were recruited together with their siblings and control infants, age- and gender-matched. The demographic data of autistic children included in this study indicates that most patients were male, similarly to that previously reported by other authors [8,9,10]. The determination of the fecal-free amino acids profile by ultra-high-performance liquid chromatography (UHPLC) with an in-house developed method showed clear differences among the three groups of children enrolled in the study. The ASD group presented higher concentrations of total amino acids as well of 14 out of the total individual amino acids; whereas, biogenic amines, GABA, and ammonium did not differ significantly among groups. It is known that glutamate can serve as a donor of amino groups in transamination reversible reactions for the synthesis of new amino acids from α-ketoacids (α-ketoglutarate, oxaloacetate, and pyruvate). We have found higher ratios of several amino acids with respect to glutamate in feces of the ASD group than in healthy children; which, could point to an imbalance in transamination reactions at the intestinal level related with autism. Differences in plasma, fecal, and urine compounds derived from the metabolism of amino acids have been previously reported between ASD and typically developing populations [7,11,12]; some of the molecules found at higher levels in ASD fecal samples were found, conversely, at reduced levels in ASD plasma samples [12]. Nevertheless, whereas De Angelis et al. [7] reported higher concentrations of most amino acids in feces of ASD children as compared to controls, other authors found lower levels of several amino acids in the feces of ASD [8,10]. Factors such as the low sample size in some of these studies, different gender distribution in the sample, different ranges of ages in the studied populations, and differences in the analytical methods employed, could account for discrepancies among relative abundance of amino acids and metabolically derived compounds found between healthy controls and patients in the different studies.

Dietary proteins and peptides are the major sources of intestinal amino acids, and a smaller proportion of these compounds may be contributed by endogenous cellular and metabolic activity of the host. Possible causes for a higher concentration of fecal amino acids in autists might be related with alterations in the digestion process and with intestinal malabsorption [13] or with shifts on colonic microbial composition. In this regard, higher relative abundance of the proteolytic genera *Bacteroides* and *Clostridioides* and lower abundance of *Faecalibacterium* have been reported in feces of ASD as compared to healthy controls [6,7,8]. Dysbiosis is often associated with an increased intestinal permeability to amino acids and peptides of dietary origin, or to microbial neurotoxic compounds. Glutamate and GABA are the major excitatory and inhibitory neurotransmitters, respectively, in the central nervous system, and GABA is generated from glutamate by a decarboxylase enzyme. An equilibrium between glutamatergic and GABAergic synapses is required for the correct functioning and development of brain, and it has been postulated that a sustained disturbed balance between these two neurotransmission ways could occur in autism [14,15,16]. We found significantly higher levels of glutamate in feces of ASD children than in samples from SIB and GP; which, was in good agreement with previous reports from other authors [5,7,17] as well as with the altered metabolism of glutamate-derived metabolites described in the gut of ASD children [12,17,18]. However, we did not evidence in our study significant differences for GABA. Although a causal relationship has not been proven to date, the influence of abnormal levels of intestinal glutamate and other neurotransmitter amino acids and their role in ASD deserve deeper research integrating the intestinal dysbiosis associated to this pathology [19,20].

Valine, leucine, isoleucine, and phenylalanine are essential amino acids whose main source are meat, fish, eggs, milk, grains, and beans; their levels were altered in our ASD children population. Phenylalanine is the precursor of tyrosine and of the mono-amine neurotransmitters dopamine, adrenaline, and noradrenaline whereas valine, leucine, and isoleucine influence brain function by modifying large neutral amino acid transport at the blood–brain barrier [21]. In this way, alterations in the metabolism and blood–brain transport of branched-chain amino acids have been recently linked with autism and other mental diseases [21,22,23,24].

One of the possible confounding factors when evaluating levels of amino acids and derived compounds is the different range of ages of the studied populations. It is known that the microbiota is influenced by age [25] and that symptoms of autism could evolve as well [26,27]; hence, when comparisons are made among individuals of different ages, this may prove difficult to extract sound conclusions. Therefore, we have analyzed, separately, differences in fecal metabolites by stratifying our sample in two ranges of ages. We found alterations for a higher number of amino acids in younger (eight amino acids) than in older children, for which only glutamine, a precursor of glutamate, glycine, and alanine levels appeared differentially increased in the ASD group with respect to control groups at the age of 8–10 years. No studies are currently available about alterations of the fecal amino acid profiles in ASD as a function of age. However, the current knowledge indicates an early onset, although subjected to some variability, of the first autistic symptoms [26,27]; which, allows hypothesizing about some differential metabolic alterations early in life related with irreversible neurological damage associated with ASD.

Regarding the severity of autism, the only difference found by us was a significantly decreased level of histidine in subjects displaying higher severity of the disease. Histidine being an essential amino acid only acquired through diet (mainly from animal sources), alterations in its levels could be related, among other possible causes, with dietary deficiencies in this group of individuals. Some researchers have found alterations in fecal and/or plasma levels of several amino acids and their derived metabolites as related with the severity of autism [7,12,16] and, specifically, fecal histidine levels were correlated with ADI-R behavioral test scores [12].

The discriminant analysis performed (LDA) allowed a good differentiation based on the fecal profile of amino acids among the three groups of children analyzed in the present work at early ages (4 to 7 years), the ASD group appearing clearly distant from healthy SIB and GP. However, amino acids were not useful to discriminate groups at a more advanced age (8 to 10 years), which indicates that the early differentiation of the ASD groups from healthy children based on the fecal profile of these compounds decreases progressively with age. On the other hand, the complexity of discriminant functions generated by the analysis precludes considering a particular compound or a reduced group of them as possible biological markers of the disease. In spite of this, the non-essential amino acids alanine, glutamine, and tyrosine had a slightly higher contribution than other compounds to separate ASD from healthy children (GP and SIB), which supports the strong metabolic basis of this disease [12]

To the best of our knowledge, this is the first study analyzing fecal amino acids, ammonium, biogenic amines, and gamma-aminobutyric acid (GABA) in Tunisian autistic children. Few studies are still available investigating the association between these and other related metabolites and autism. Another strength of our study, which supports the interpretation of results, is the selection of two control groups, one related (SIB) and one unrelated (GP) to the ASD group. Nevertheless, this work has potential limitations, as it does not include the assessment of dietary habits and given the limited sample size. Further efforts are needed to elucidate the role that early metabolic alterations could play in the development and prognosis of ASD. In the same way, the surveillance of children with early atypical behaviors that may involve augmented risk of autism development is of paramount importance. Personalized diets, designed based on their nutrients content, which pay special attention to the amino acids profile of protein sources, could be applied in early stages of autism development to prevent or minimize cognitive impairment and neurodevelopment dysfunctions associated with this disease.

## 4. Materials and Methods

### 4.1. Subjects and Study Design

The human sample included 74 children from 4 to 10 years old, distributed into three groups: 28 autistic children (22 males and 6 females), 18 brothers and sisters (siblings) in the same range of ages (13 female and 5 males), and 28 children from the general population that were matched with autists by gender, age, socio-economic status, and geographic region (22 males and 6 females). Autistic children were recruited at the Unit of Child and Adolescent Department of Psychiatry from the Fattouma Bourguiba University Hospital in Monastir (Tunisia) between 2019 and 2020 among children attending for consultation. Autistic children were diagnosed following the fifth edition of the Diagnostic and Statistical Manual of Mental Disorders (DSM-5) [28], the Autism Diagnostic Inventory-Revised (ADI-R) [29], and the Autism Diagnostic Observation Schedule-2 (ADOS-2) [30]. The severity of autism was determined according to the Childhood Autism Rating Scale, 2nd edition (CARS) [31,32] for the severity of the disease into two groups: mild to moderate and severe. The inclusion criteria for the ASD group were children aged between 4 and 10 years and diagnosed with ASD according to international established and validated tools (DSM-5, ADOS-2, ADI-R and CARS). As for SIB and GP groups, we included children with the same range of age as the ASD group who did not show signs of developmental disorders or psychiatric diseases. The exclusion criteria for the three groups were having suffered infections, antibiotics or anti-fungal treatment or having consumed probiotics and/or prebiotics in the previous month of recruitment, unbalanced or special diets, other neurological disorders not associated with autism, type 1 diabetes, genetic syndromes, celiac disease, food intolerance, or inflammatory bowel disease.

The protocol of this observational study was approved by the Medical Ethical Committee of the Faculty of Medicine of Monastir. Tunisia (reference IORG 0009738 Nº18/OMB 0990-0279) and by the Bioethics Committee of CSIC (reference 172/2020). The participation in this study was voluntary, and parents provided a signed informed consent for the participation of their children. The privacy of participating children was protected.

### 4.2. Fecal Sample Collection, Transportation and Preparation of Cell-Free Supernatants

Fecal samples were obtained from volunteers participating in the study. Samples were collected in sterile plastic containers after deposition, immediately frozen at −20 °C, and transported to the laboratory of Transmissible Diseases and Biologically Active Substances at the Faculty of Pharmacy in Monastir (Tunisia). Fecal samples were further imported by the laboratory of IPLA-CSIC (Villaviciosa, Spain) frozen on dry ice, following international rules for transportation of biological substances. For analyses, fecal samples were thawed, and 1/10 (*w*/*v*) dilution was prepared in phosphate buffered saline (PBS) and homogenized in a Lab-Blender 400 stomacher (Seward Medical, London, UK) for 5 min. Cell-free supernatants were obtained by centrifugation and used for chromatographic analyses.

### 4.3. Chromatographic Analyses of Amino Acids and Amino-Derived Compounds

Quantification of amino acids, derived biogenic amines, ammonium, and GABA was carried out by UHPLC following a derivatization with diethyl ethoxymethylenemalonate (DEEMM, Sigma-Aldrich, St. Louis, MO, USA) in 100 µL of fecal water, by the procedure described in Redruello et al. [33], adapted and optimized to human fecal cell-free supernatants by Salazar et al. [34]. Samples were filtered through 0.22 µm pore diameter PTFE membranes (VWR International, Radnor, PA, USA) prior to injection of 1 µL into the UHPLC chromatographic system. Concentrations of the analyzed compounds in fecal supernatants were calculated in millimolar (mM) and referred as concentration in fecal samples.

### 4.4. Statistical Analysis

Statistical analysis of results was performed using the software SPSS v.26 (SPSS Inc., Chicago, IL, USA). Data for the different compounds analyzed were compared among fecal samples from the three groups of children (ASD, SIB, and GP) considered as such or stratified by age (4 to 7 years, 8 to 10 years). Ammonium, the only variable displaying a normal distribution (Shapiro–Wilk test), was analyzed by one-way ANOVA. For the remaining compounds a Kruskal–Wallis test followed by a post hoc Dunn’s test were applied. For two groups comparison in children diagnosed with ASD, between those displaying mild-to-moderate and those showing severe symptoms, a two-tailed Student’s t-test (for ammonium) or U-Mann Whitney test (for the remaining variables) were conducted for the evaluation of data by parametric or non-parametric contrast, respectively. Unless otherwise specified, data of numerical variables are presented as mean ± standard deviation. Frequencies comparison of categorical variables related to demographic characteristics of the population was performed by a Chi-Square test or Fisher’s exact test. A significant *p* value of 0.05 was used for the interpretation of results. Figures were constructed with GraphPad PRISM (version 8.0.2)

LDA was carried out with the XLSTAT 2022 software (Addinsoft, Longueuil, Canada) in order to clarify whether differences among groups of children could be established on the basis of fecal amino acid profiles. The three groups of children (ASD, SIB, and GP) were considered as the categorical grouping variable and fecal levels of the different amino acids were used as predictor variables. Analyses were performed separately for children of 4–7 and 8–10 years of age and considering only the variables with concentration values higher than the quantitation limit of the method.

## Figures and Tables

**Table 1:** General demographic characteristics of the study population. Values showing different letters (^a, b^) at the right side indicate significant differences (*p*-value < 0.05) in gender among the different groups of children. ASD, Autism Spectrum Disorder; SIB, Siblings; GP, General Population. CARS: Childhood Autism Rating Scale, 2nd edition.

	ASD % (n)	SIB % (n)	GP % (n)
Gender			
Male	78.6 (22) ^a^	27.8 (5) ^b^	78.6 (22) ^a^
Female	21.4 (6) ^b^	72.2 (13) ^a^	21.4 (6) ^b^
Age			
4–7 years	35.7 (10)	61.1 (11)	50 (14)
8–10 years	64.3 (18)	38.9 (7)	50 (14)
Severity (CARS score)			
mild to moderate (score 30–36)	39.3 (11)	-	-
severe (score 37–60)	60.7 (17)	-	-

## Data Availability

Not applicable.

## Supplementary Materials

The following supporting information can be downloaded at: https://www.mdpi.com/article/10.3390/molecules28073269/s1, Figure S1: Fecal levels of gamma aminobutyric acid, biogenic amines and ammonium in samples from autistic children, their siblings and children from the general population; Figure S2: Fecal levels of gamma aminobutyric acid, biogenic amines and ammonium in samples from autistic children, their siblings and children from the general population stratified by age; Figure S3: Fecal levels of gamma aminobutyric acid, biogenic amines and ammonium in samples from autistic children, their siblings and children from the general population, analyzed according to the severity of disease; Table S1: Fecal levels of total and free amino acids grouped by the type of side chain; Table S2: Correlations of variables used in the linear discriminant analysis to the discriminant functions at the age of 4–7 years; Table S3: Correlations of variables used in the linear discriminant analysis to the discriminant functions at the age of 8–10 years.
