# Supplementary material for: An Overview on Fecal Profiles of Amino Acids and Related Amino-Derived Compounds in Children with Autism Spectrum Disorder in Tunisia
Source: Molecules. 2023 Apr 6;28(7):3269. doi: 10.3390/molecules28073269 (PMC10096484; doi:10.3390/molecules28073269)
Supplement: Supplementary file 1 [file molecules-28-03269-s001.zip › Figure S1.pdf]

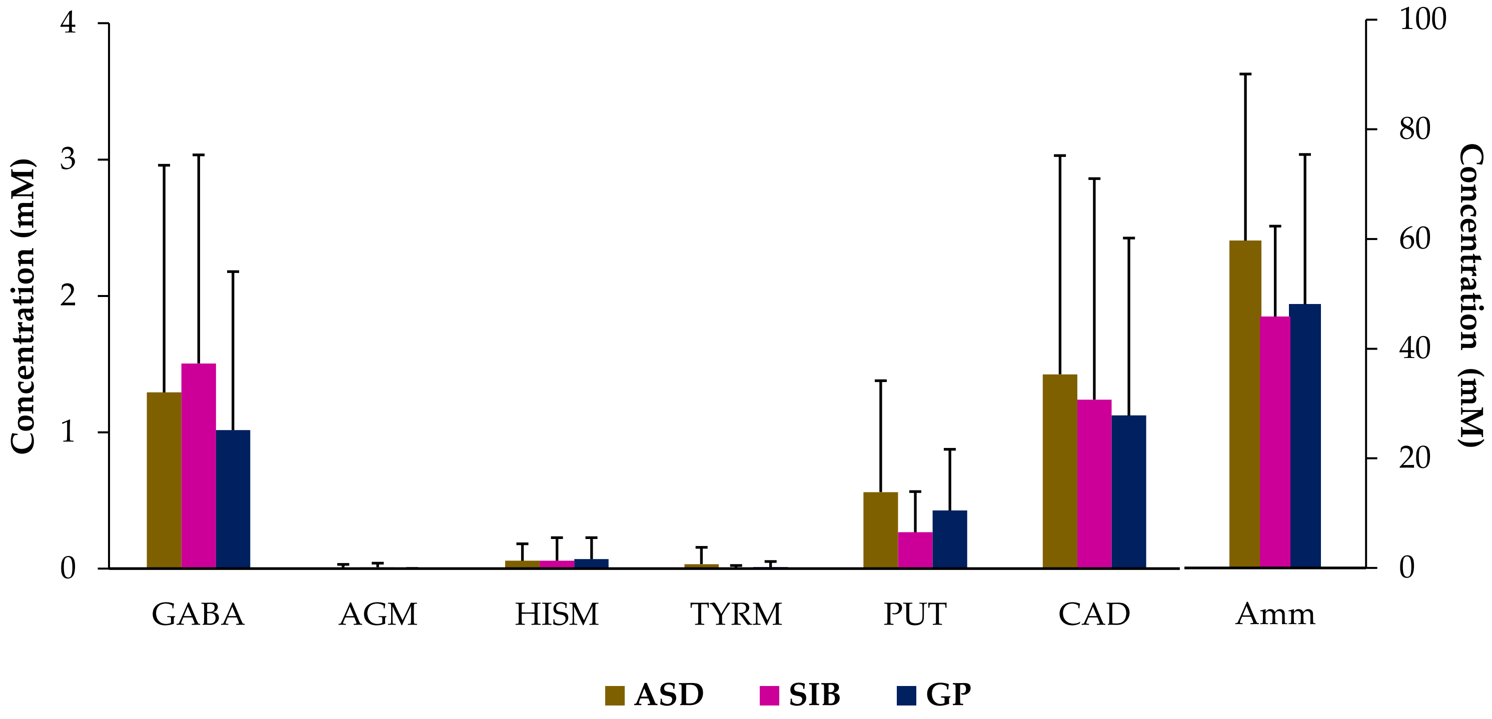

Figure S1. Fecal levels of gamma aminobutyric acid, biogenic amines and ammonium in samples from autistic children, their siblings and children from the general population. Bars represent mean values and vertical lines on the bars represent standard deviation. GABA: gamma aminobutyric acid; AGM: agmatine; HISM: histamine; TYRM: tyramine; PUT: putrescine; CAD: cadaverine; Amm: ammonium. ASD: autism spectrum disorder; SIB: siblings; GP: children from the general population
