# Supplementary material for: An Overview on Fecal Profiles of Amino Acids and Related Amino-Derived Compounds in Children with Autism Spectrum Disorder in Tunisia
Source: Molecules. 2023 Apr 6;28(7):3269. doi: 10.3390/molecules28073269 (PMC10096484; doi:10.3390/molecules28073269)
Supplement: Supplementary file 1 [file molecules-28-03269-s001.zip › Figure S2.pdf]

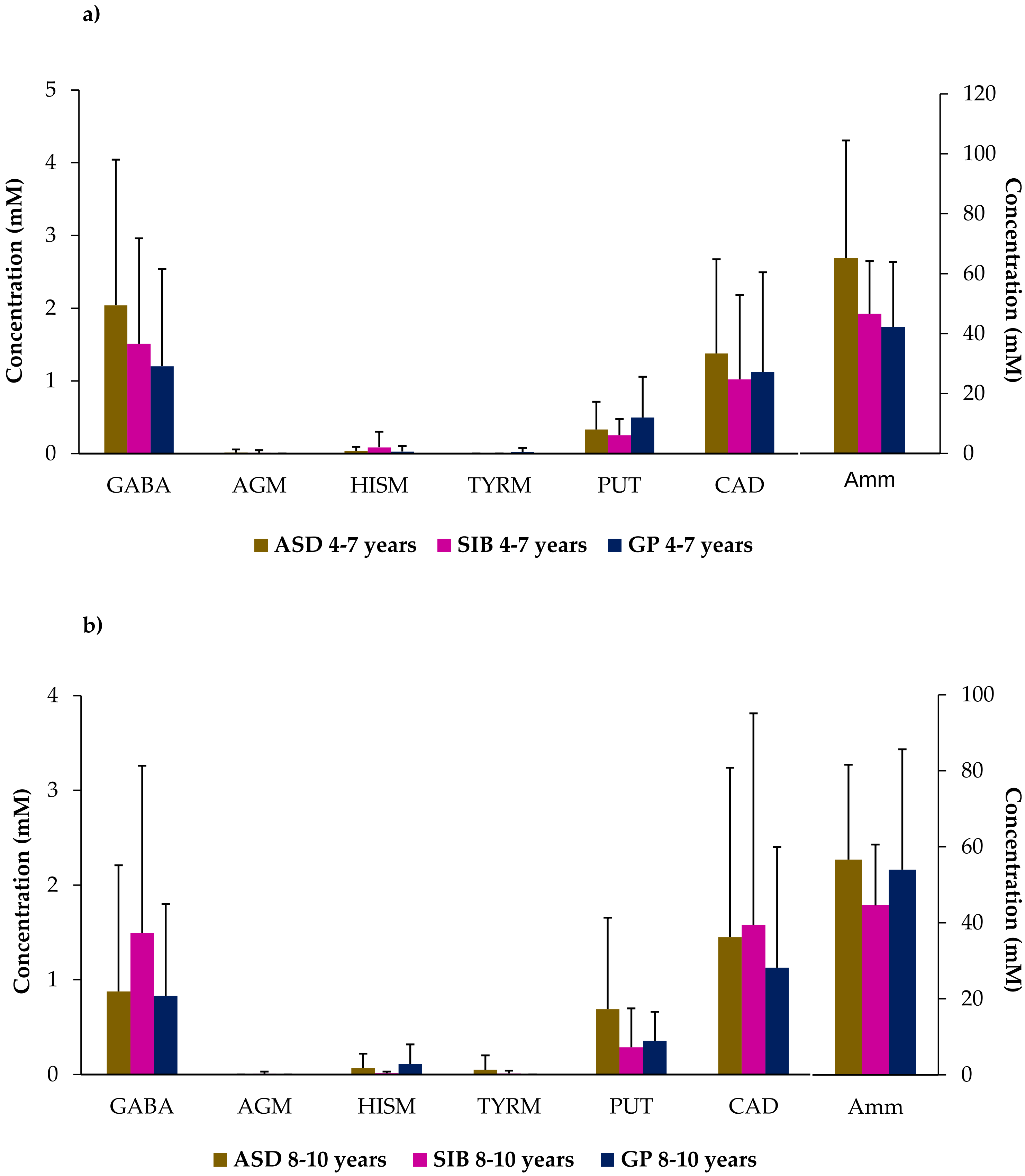

Figure S2. Fecal levels of gamma aminobutyric acid, biogenic amines and ammonium in samples from autistic children, their siblings and children from the general population stratified by age: (a) 4-7 years. (b) 8-10 years. Vertical lines on the bars represent standard deviation. GABA: gamma aminobutyric acid; AGM: agmatine; HISM:

histamine; TYRM: tyramine; PUT: putrescine; CAD: cadaverine; Amm: ammonium. ASD: autism spectrum disorder; SIB: siblings; GP: children from the general population
