# Supplementary material for: An Overview on Fecal Profiles of Amino Acids and Related Amino-Derived Compounds in Children with Autism Spectrum Disorder in Tunisia
Source: Molecules. 2023 Apr 6;28(7):3269. doi: 10.3390/molecules28073269 (PMC10096484; doi:10.3390/molecules28073269)
Supplement: Supplementary file 1 [file molecules-28-03269-s001.zip › Figure S3.pdf]

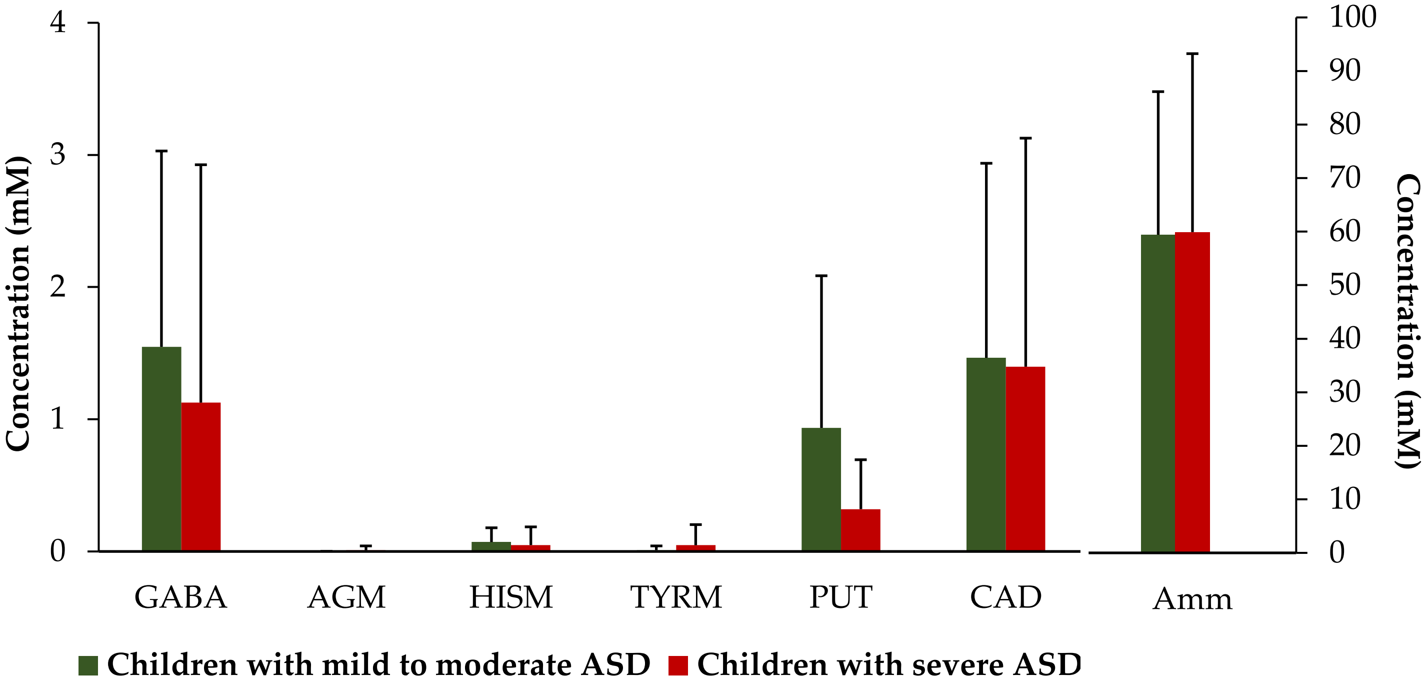

Figure S3. Fecal levels of gamma aminobutyric acid, biogenic amines and ammonium in samples from autistic children analyzed according to the severity of disease**.** Vertical lines on the bars represent standard deviation. GABA: gamma aminobutyric acid; AGM: agmatine; HISM: histamine; TYRM: tyramine; PUT: putrescine; CAD: cadaverine; Amm: ammonium. ASD: autism spectrum disorder. The severity of the disease was determined according to to CARS score: mild to moderate and severe
