# Supplementary material for: An Overview on Fecal Profiles of Amino Acids and Related Amino-Derived Compounds in Children with Autism Spectrum Disorder in Tunisia
Source: Molecules. 2023 Apr 6;28(7):3269. doi: 10.3390/molecules28073269 (PMC10096484; doi:10.3390/molecules28073269)
Supplement: Supplementary file 1 [file molecules-28-03269-s001.zip › Table S1.pdf]

Table S1. Fecal levels of total and free amino acids grouped by the type of side chain: branched-chain (leucine, isoleucine and valine), aromatic (tyrosine, phenylalanine and tryptophan) and aliphatic (alanine, valine, leucine, isoleucine, and glycine). Concentrations (mM) in fecal samples of autistic children (ASD), their siblings (SIB) and children from the general population (GP) considered jointly or disaggregated by age: 4-7 years, and 8-10 years. Data are expressed as mean ± standard deviation of two independent determinations per sample. Different letters to the right of the values indicate significant differences among the three groups of children ($p < 0.05$).

**Amino acid group**	**Whole sample**			**4-7 years**			**8-10 years**		
	**ASD**	**SIB**	**GP**	**ASD**	**SIB**	**GP**	**ASD**	**SIB**	**GP**
Total	49.99 ± 22.59 **a**	35.67 ± 16.98 **b**	29.57 ± 10.95 **b**	57.10 ± 23.64 **a**	38.53 ± 18.43 **ab**	29.25 ± 8.58 **b**	46.04 ± 21.64 **a**	31.16 ± 14.57 **ab**	29.88 ± 13.24 **b**
Branched	9.59 ± 4.91 **a**	6.92 ± 3.24 **ab**	6.01 ± 2.25 **b**	10.98 ± 5.13	7.42 ± 3.41	6.18 ± 2.12	8.81 ± 4.76	6.14 ± 3.02	5.84 ± 2.45
Aromatics	4.16 ± 2.04 **a**	2.99 ± 1.43 **ab**	2.56 ± 0.94 **b**	4.91 ± 1.96 **a**	3.23 ± 1.60 **ab**	2.52 ± 0.70 **b**	3.75 ± 2.02	2.60 ± 1.11	2.601 ± 1.161
Aliphatics	26.51 ± 11.95 **a**	18.51 ± 9.40 **b**	14.24 ± 6.21 **b**	30.29 ± 13.11 **a**	20.00 ± 9.92 **ab**	14.29 ± 5.58 **b**	24.41 ± 11.08 **a**	16.17 ± 8.7 **ab**	14.20 ± 6.99 **b**
