# Supplementary material for: An Overview on Fecal Profiles of Amino Acids and Related Amino-Derived Compounds in Children with Autism Spectrum Disorder in Tunisia
Source: Molecules. 2023 Apr 6;28(7):3269. doi: 10.3390/molecules28073269 (PMC10096484; doi:10.3390/molecules28073269)
Supplement: Supplementary file 1 [file molecules-28-03269-s001.zip › Table S2.pdf]

Table S2. Correlations of variables used in the linear discriminant analysis to the discriminant functions at the age of 4-7 years, based on levels of 19 amino acids determined in fecal samples of autistic children, their siblings and children from the general population.

Metabolite	F1	F2
Aspartate	0,475	-0,067
Glutamate	0,486	0,045
Asparagine	0,161	0,259
Serine	0,388	-0,035
Glutamine	0,599	0,100
Histidine	0,307	0,143
Glycine	0,536	0,056
Threonine	0,436	0,085
Arginine	0,365	-0,123
Alanine	0,669	-0,094
Tyrosine	0,662	0,046
Valine	0,531	0,071
Methionine	0,487	0,006
Tryptophan	0,549	0,056
Isoleucine	0,508	0,058
Leucine	0,527	0,019
Phenylalanine	0,536	-0,012
Ornithine	0,419	0,034
Lysine	0,517	0,011
