# Supplementary material for: An Overview on Fecal Profiles of Amino Acids and Related Amino-Derived Compounds in Children with Autism Spectrum Disorder in Tunisia
Source: Molecules. 2023 Apr 6;28(7):3269. doi: 10.3390/molecules28073269 (PMC10096484; doi:10.3390/molecules28073269)
Supplement: Supplementary file 1 [file molecules-28-03269-s001.zip › Table S3.pdf]

Table S3. Correlations of variables used in the linear discriminant analysis to the discriminant functions at the age of 8-10 years, based on levels of 19 amino acids determined in fecal samples of autistic children, their siblings and children from the general population.

Metabolite	F1	F2
Aspartate	-0,283	0,177
Glutamate	-0,251	0,470
Asparagine	-0,287	-0,017
Serine	-0,194	0,156
Glutamine	-0,413	0,133
Histidine	-0,311	0,332
Glycine	-0,448	0,295
Threonine	-0,268	0,207
Arginine	-0,113	-0,042
Alanine	-0,624	0,223
Tyrosine	-0,346	0,256
Valine	-0,412	0,254
Methionine	-0,385	0,227
Tryptophan	-0,377	0,206
Isoleucine	-0,385	0,231
Leucine	-0,393	0,218
Phenylalanine	-0,371	0,263
Ornithine	-0,218	0,239
Lysine	-0,388	0,199
